# The Effect of CO_2_ Laser Treatment on the Composition of Cotton/Polyester/Metal Fabric

**DOI:** 10.3390/polym18020215

**Published:** 2026-01-13

**Authors:** Andris Skromulis, Inga Lasenko, Imants Adijāns, Ilze Liepiņlauska, Maido Merisalu, Uno Mäeorg, Svetlana Sokolova, Sandra Vasilevska, Sai Pavan Kanukuntla, Jaymin Vrajlal Sanchaniya

**Affiliations:** 1 Engineering Center, Riga Technical University–Rezekne Academy, Atbrīvošanas Aleja 76, LV-4601 Rezekne, Latvia; 2 Institute of Mechanical and Biomedical Engineering, Riga Technical University, Ķīpsalas iela 6A, LV-1048 Rīga, Latvia; 3 Institute of Physics, University of Tartu, 50411 Tartu, Estonia; 4 Institute of Chemistry, University of Tartu, 50411 Tartu, Estonia

**Keywords:** CO_2_ laser treatment, cotton/polyester/metal hybrid fabric, laser-induced morphology modification, mechanical property degradation

## Abstract

The effect of CO_2_ laser treatment on the surface composition and properties of a woven fabric (polyester (PET) fiber (59 wt%)/cotton (CO) fiber (31 wt%)/stainless-steel (SS) metal fibers (10 wt%)) was investigated across a range of laser intensities (19.1 × 10^6^ to 615.0 × 10^6^ W/m^2^). Elemental analysis using wavelength-dispersive X-ray fluorescence (WD-XRF) revealed that for an intensity up to 225.4 × 10^6^ W/m^2^, the carbon content on the fabric surface increased while the oxygen content decreased, indicating thermally induced surface modification. Fourier transform infrared (FT-IR) spectroscopy confirmed that no new chemical bonds were formed, suggesting that the changes observed were predominantly physical in nature. High-resolution scanning electron microscopy (HR-SEM) showed progressive fiber fusion and surface smoothing with increasing laser intensity, consistent with polyester melting. Tensile testing demonstrated a significant decline in peak load and elongation at peak load with rising laser fluence, indicating mechanical embrittlement. Overall, CO_2_ laser treatment alters the morphology and elemental composition of the fabric surface without inducing major chemical decomposition, markedly reducing its mechanical strength.

## 1. Introduction

Cotton/polyester blended fabrics are widely used in military applications due to their balanced combination of durability, breathability, and comfort. These blends are commonly employed in the production of battle dress uniforms, combat trousers, tents, backpacks, and covers, where both mechanical resilience and environmental adaptability are critical. Additionally, these fabrics are also valued for their compatibility with camouflage printing, an essential requirement in operational environments [1,2]. The mechanical behavior of polymer-based textiles depends fundamentally on fiber-level properties and their integration into bulk structures. Recent advances in nanofiber reinforcement have demonstrated significant potential for enhancing composite mechanical properties [3,4], while thermal treatment processes can substantially alter fiber characteristics and performance [5].

In high-performance textile applications, surface modification plays a vital role in enhancing fabric performance, whether for functional coatings, infrared camouflage, or reduced detectability. However, conventional surface finishing techniques often rely on wet chemical treatments that can be environmentally hazardous and difficult to control uniformly [6]. These methods also present logistical challenges in field-ready or low-resource settings. Understanding microstructure–property relationships in hybrid fabrics is particularly crucial, as advanced characterization methods, including finite element modeling, have revealed that single-fiber modifications significantly influence aggregate mechanical behavior [7]. Recent advances in nanofiber reinforcement have demonstrated significant potential for enhancing composite mechanical properties [3], while thermal treatment processes can substantially alter fiber characteristics and performance [5]. Moreover, comprehensive physical–mechanical assessment protocols are essential for technical textile applications where performance optimization is critical [8]. Despite these advantages, a systematic understanding of how laser intensity affects elemental composition, chemical structure, and mechanical properties simultaneously remains limited, particularly for blended fabrics containing metallic fibers.

Laser treatment, particularly using CO_2_ lasers, has emerged as a promising alternative for clean, contactless surface modification of textiles [9,10,11,12]. This technique enables precise structural and chemical modification of polymer-containing fabrics, including polyester/cotton blends, without the use of solvents or chemical reagents [13,14,15,16,17,18]. CO_2_ laser irradiation can induce localized melting, fiber fusion, and surface restructuring, offering potential improvements for fabric performance and enabling downstream functionalization.

Military textiles, particularly those incorporating metal fibers for EMI (electromagnetic interference) shielding, must meet rigorous standards for durability and protective performance. Current technologies often rely on metal coatings or chemical finishes which suffer from low breathability, potential skin irritation, and significant degradation after washing or mechanical stress. Furthermore, traditional wet chemical treatments (like caustic reduction) are difficult to control locally and can weaken the entire fabric structure. Besides chemical and laser treatments, other methods such as plasma treatment, vacuum deposition (sputtering), and electroless plating are used for textile modification [19]. However, these methods often involve high costs or environmentally hazardous precursors. While these methods exist, CO_2_ laser treatment offers a unique advantage in localized, dry, and high-speed processing of hybrid metal-polymer systems. It allows for controlled surface melting of the thermoplastic component (polyester), which can ‘lock’ the metal fibers into the fabric matrix, potentially enhancing the durability of the shielding effect without the environmental and structural downsides of bulk chemical processing. The interaction between CO_2_ laser radiation and polymer-based textiles is a complex photothermal process. According to Katzir et al. [20], IR laser radiation induces significant changes in the absorption spectra of thermoplastic polymers due to localized thermal effects that can lead to phase transitions or molecular reorganization without necessarily forming new chemical species. In thermoplastic materials like polyethylene terephthalate (PET), the absorption of IR energy at specific wavelengths (typically around 10.6 μm for CO_2_ lasers) results in rapid heating, which can modify the crystallinity and surface energy of the fibers. Understanding these fundamental spectral changes is crucial for interpreting the stability of the chemical bonds observed in the subsequent FT-IR analysis of laser-treated fabrics.

Previous studies have investigated the influence of CO_2_ laser treatment on cotton and blended textiles. For example, On-na Hung et al. [21,22] demonstrated that laser exposure causes partial melting of the polyester component, leading to increased fabric stiffness. In another study, On-na Hung et al. [23] proved with a solubility test that laser treatment etched cotton fibers away in cotton/polyester fabric. At the same time, molten polyester re-solidified on the yarn. In another study, On-na Hung et al. showed that CO_2_ laser treatment of the fabric prior to dying reduces dye intake and results in a lighter color [24]. However, these studies focused primarily on pure cotton or simple blends without metallic components. Comprehensive correlation between laser intensity, surface elemental composition (via XRF), morphological changes, and mechanical degradation across a wide intensity range (19.1–615.0 × 10^6^ W/m^2^) has not been investigated for cotton/polyester/stainless-steel hybrid fabrics.

This work addresses this gap by providing the first systematic multi-technique characterization (XRF, FT-IR, HR-SEM, and tensile testing) of laser-intensity-dependent changes in hybrid textiles containing metallic fibers. The aim of this study is to investigate the effect of CO_2_ laser treatment on 31/59 cotton/polyester fabric, integrating 10% stainless-steel metal staple fibers, across a range of laser intensities. Through a combination of high-resolution scanning electron microscopy (HR-SEM), wavelength-dispersive X-ray fluorescence (WD-XRF), Fourier transform infrared spectroscopy (FT-IR), and tensile testing, we aim to assess how laser treatment alters the surface composition and structure of the fabric. Our findings are expected to inform the development of laser-based surface processing techniques for next-generation military textiles, enabling improved material performance, durability, and their integration with protective and electronic systems.

## 2. Materials and Methodology

The fabric used in this study was a hybrid woven textile composed of blended yarns containing three components: conventional polyester (PET) fiber (59 wt%), cotton (CO) fiber (31 wt%), and BEKINOX stainless-steel (SS) metal staple fibers (10 wt%). Both the PET and cotton fibers were white in color. The stainless-steel fibers had a diameter of 8 μm and an average fiber length of 45 mm. The material was purchased from the SIA TEXREP BALTICA (Rīga, Balasta dambis 80A, LV-1048).

The three types of fiber components (PET, CO and SS) were mixed at the drawing frame, and a ring spinning system was used to produce blended single yarns with a linear density of 30 tex. The hybrid fabric was manufactured using these conductive yarns in a 2/1 twill weave structure, with 100% hybrid yarn placement in both warp and weft directions. The warp sett was 39 ends/cm, and the weft sett was 22 picks/cm. The fabric had a thickness of 0.36 mm and a mass per unit area of 190 g/m^2^.

Laser treatment was performed using an infrared CO_2_ laser (λ = 10,640 nm; ST-CC9060, Suzhou Suntop Laser Technology Co., LTD, Suzhou, China) operating in continuous-wave (CW) mode. Fifteen different laser power densities (I, W/m^2^) were applied (Table 1), using a scanning speed of 100 mm/s and a laser spot diameter of 0.1 mm. Laser processing was conducted in a zigzag (hatch) pattern with 0% line overlap. Table 1 shows the intensities used to treat the 15 fabric samples.

The available laser power setting ranges from 1% to 100%. In this study, the laser power was adjusted by 0.1% increments. For most consecutive samples, a 0.1% increase in laser power led to an intensity increase between 21,7·10^6^ W/m^2^ and 38,2·10^6^ W/m^2^ (on average 28,4·10^6^ W/m^2^). A pronounced increase in intensity was observed between samples 12 and 13, where intensity reached 226,8·10^6^ W/m^2^ (for a feature of the laser equipment at a given level of intensity).

Laser processing was conducted in a zigzag (hatch) pattern with 0% line overlap.

To evaluate changes in color on the surface of the material (after laser treatment with varying intensities), a colorimetric system based on human color perception was used. We used the CIE L*a*b* (or CIELAB) system based on the ASTM E308: Standard Practice for Computing the Colors of Objects by Using the CIE System.

Mechanical properties were evaluated in both the warp and weft directions following DIN EN ISO 13934-1:2013. Tensile testing was performed using a Zwick/Roell Z150 universal testing machine (Zwick GmbH & Co. KG, Ulm, Germany) with a preload of 1 N and a crosshead speed of 100 mm/min. For each sample, five measurements were recorded in both directions to determine the peak load at break (F_H, N), elongation at peak load (ε_H, %), breaking load (F_B, N), and elongation at breaking point (ε_B, %).

Surface morphology was characterized using high-resolution scanning electron microscopy (HR-SEM) on a Helios Nanolab 600 dual-beam system (FEI Company, Hillsboro, OR, USA). To ensure sufficient surface conductivity, the fabric samples were coated with a 12 nm layer of silver using magnetron sputtering. SEM images were acquired using 3 kV primary electrons and secondary electron detection.

Elemental analysis of the laser-treated fabric surfaces was conducted using wavelength-dispersive X-ray fluorescence (WD-XRF) spectroscopy with an AZX 400 spectrometer (Rigaku, Tokyo, Japan). The chemical composition and infrared absorption behavior were further examined by Fourier transform infrared (FT-IR) spectroscopy using a Nicolet 6700 FT-IR spectrometer (Thermo Scientific, Waltham, MA, USA), equipped with a Smart Orbit diamond micro-ATR accessory (refractive index = 2.4; active area diameter = 1.5 mm; angle of incidence = 45 °C).

The diffuse reflectance of visible light from the fabric surface was measured using a Jasco (Tokyo, Japan) V-570 UV-Vis-NIR spectrophotometer operated at near-normal incidence geometry. The reflection angle, defined as the angle between the reflected light beam and the surface, was maintained near 0 °C to minimize specular reflection. Reflectance values were recorded relative to a calibrated reference mirror, which was assigned 100% reflectance. All measurements were conducted in the visible wavelength range (420–690 nm) under identical illumination and alignment conditions.

The surface wettability was characterized by measuring the static water contact angle (WCA) using the sessile drop method. A high-resolution camera with a macro lens was used to capture the profile of a 5 μL droplet of distilled water (ISO 3696, type 2, Analytical, <1.0 µS/cm, General chemical analysis) deposited onto the fabric surface. For each sample, five measurements were performed at different surface locations to ensure reproducibility. The contact angles θ were calculated by analyzing the drop profile images using ImageJ software, v1.54r (to calculate the precise contact angle values) by drawing a tangent line at the liquid-solid-air triple point. All tests were conducted under standard laboratory conditions (20 ± 2 °C and 65 ± 5% relative humidity. To ensure statistical reliability, measurements were performed at five different locations on each sample, and the results were reported as the mean value ± standard deviation.

## 3. Results and Discussion

### 3.1. Laser Treatment Effect on Color

**Figure 1:**
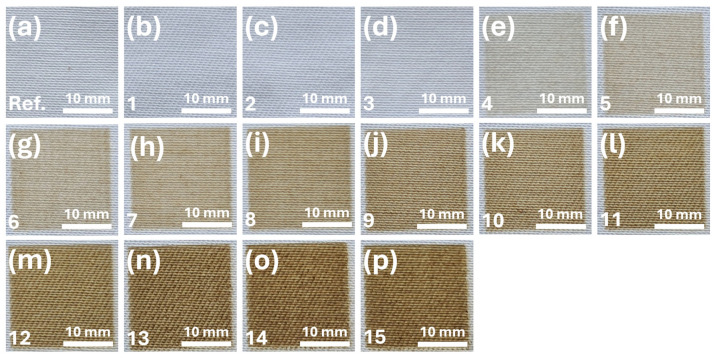
Photographs of white cotton/polyester/metal fabric before and after CO_2_ laser treatment at different intensities (samples 1–15). Visible surface changes correspond to increasing laser power, ranging from 19.1 × 10^6^ to 615.0 × 10^6^ W/m^2^. Subfigures (**a**–**p**) correspond to samples listed in Table 1 in order of increasing laser intensity.

Photographs of the untreated and CO_2_-laser-treated cotton/polyester/metal fabric samples are presented in Figure 1. The images presented in Figure 1 show that laser treatment in the intensity range of 19.1 × 10^6^ to 615.0 × 10^6^ W/m^2^ can be used to effectively modulate the visual appearance of fabric, particularly its level of darkness. As laser intensity increases, the fabric gradually exhibits a darker hue, indicating thermal surface modification or partial carbonization. The first noticeable change in color was observed at an intensity of 121.0 × 10^6^ W/m^2^ (Figure 1e) (according to the ASTM E308: Standard Practice for Computing the Colors of Objects by Using the CIE System), suggesting a threshold effect. Beyond 554.0 × 10^6^ W/m^2^ (Figure 1n), further darkening appeared to plateau, indicating a saturation point in color change induced by a laser.

The cotton/polyester/metal fabric samples darkened progressively with increasing laser power, transitioning from an initial white (reference) to yellowish and eventually brown hues. To investigate this color transformation more quantitatively, UV-Vis diffuse reflectance spectra were collected from three representative samples: the untreated white reference (Figure 1a), lightly modified sample 4 (Figure 1e, irradiated at 121 × 10^6^ W/m^2^), and significantly darkened sample 14 (Figure 1o, irradiated at 586 × 10^6^ W/m^2^).

The reflectance spectrum of sample 4 (Figure 1e) showed a gradual decrease in reflected intensity with shorter wavelengths, indicating increased absorption in the blue-violet region. Sample 14 (Figure 1o) exhibited the lowest overall reflectance across the measured range (420–690 nm), maintaining the same downward trend with decreasing wavelength. This indicates enhanced absorption in the visible spectrum, consistent with the observed darkening. No distinct absorption bands were present in the spectra, suggesting that the changes were primarily due to broadband absorption caused by laser-induced surface carbonization or microstructural changes, rather than the formation of specific chromophores.

**Figure 2:**
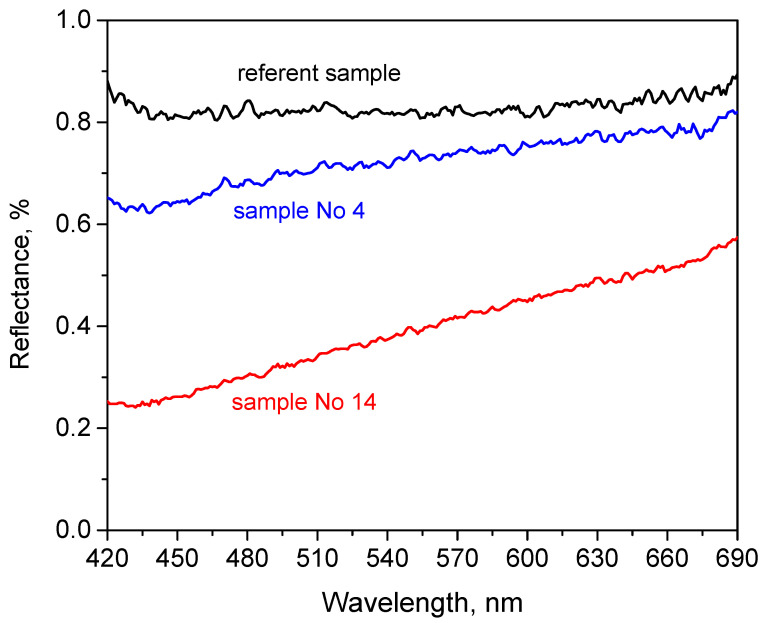
Diffuse reflectance spectra of selected cotton/polyester/metal fabric samples measured at near-normal incidence.

The reflectance is shown as a function of wavelength (420–690 nm) for the untreated reference fabric (Figure 1a), sample 4 (Figure 1e) (121 × 10^6^ W/m^2^), and sample 14 (Figure 1o) (586 × 10^6^ W/m^2^), illustrating the in visible light reflection induced by the laser. Figure 2 presents the diffuse reflectance spectra of selected cotton/polyester/metal fabric samples as a function of CO_2_ laser treatment intensity.

### 3.2. Laser Treatment Effect on Surface Morphology

**Figure 3:**
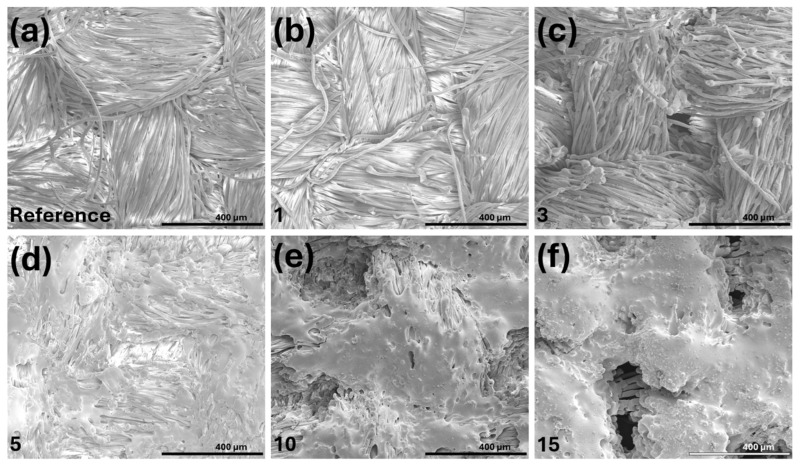
Scanning electron microscopy (SEM) secondary electron images of cotton/polyester/metal fabric samples: (**a**) untreated reference; (**b**) sample treated at laser intensity 19.1 × 10^6^ W/m^2^; (**c**) sample treated at laser intensity 89.13 × 10^6^ W/m^2^; (**d**) sample treated at laser intensity 146.4 × 10^6^ W/m^2^; (**e**) sample treated at laser intensity 273.7 × 10^6^ W/m^2^; and (**f**) sample treated at laser intensity 615.0 × 10^6^ W/m^2^.

Scanning electron microscopy (SEM) images of the untreated cotton/polyester/metal reference sample (Ref.) and laser-treated samples 1, 3, 5, 10, and 15 (corresponding to Figure 3a–f, respectively) are shown in Figure 3.

The figures were selected based on the most pronounced changes observed in the surface morphology of the material. As illustrated, increasing laser intensity progressively induces more pronounced transformations in surface morphology, consistent with thermally driven structural modification. Comparing the Ref. sample (Figure 3a) with samples 1–5 (Figure 3b–d) at lower intensities of laser treatment, the effects range from minor surface roughening in sample 1 (Figure 3b) (likely due to superficial polymer chain scission or mild thermal shrinkage) to extensive surface melting and polymer coalescence in sample 5 (Figure 3d). In this sample, individual fibers are nearly indistinguishable. In this range, the polyester component, which has a lower melting point than cotton, appears to undergo partial flow and re-solidification, resulting in fiber fusion and a significant loss of porosity. Among samples 5 to 15 (Figure 3d–f), the fibrous architecture becomes increasingly obscured as melting intensifies, producing smoother, consolidated surfaces. The larger voids observed between yarns (particularly at crossover regions) are likely due to bulk material shrinkage during melting, as the originally fluffy fabric compacts into a denser structure. These morphological changes may have critical implications for surface-dependent properties such as wettability, mechanical interlocking, and interfacial adhesion in subsequent processing steps (e.g., fabric finishing) [21].

**Figure 4:**
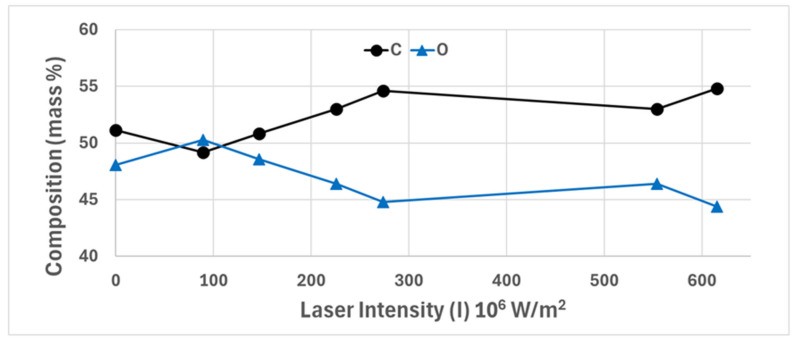
Surface elemental composition (mass % of carbon and oxygen) of cotton/polyester/metal fabric samples as a function of applied laser intensity, measured using X-ray fluorescence (XRF) spectroscopy.

As shown in Figure 3, progressive changes in surface morphology, including fiber fusion, melting, and compaction, are evident with increasing laser intensity. Based on a visual assessment of the fabric surface morphology (after laser treatment), the most uniform structure was sample (d) (Figure 3d), which correlates with the results of the XRF study shown in Figure 4.

### 3.3. Effect of Laser Treatment on Elemental Composition

As shown in Figure 4, the elemental surface composition of laser-treated cotton/polyester/metal fabrics, determined via X-ray fluorescence (XRF) spectroscopy, exhibits a systematic dependence on laser intensity. (Error bars are present but obscured by the size of the data points. Trend lines are included to guide the eye). Specifically, the atomic concentration of carbon (C) increases monotonically with rising laser fluence, while the concentration of oxygen (O) decreases correspondingly. This inverse relationship suggests a thermally induced surface modification, likely due to localized degradation or volatilization of oxygen-containing functional groups. This trend persists up to a threshold intensity of 225.4 × 10^6^ W/m^2^ (sample 8, Figure 1i), beyond which the surface concentrations of C and O plateau, indicating a saturation point in the laser-treated material interaction. This saturation behavior may be attributed to the steady-state composition attained or the formation of a carbonaceous layer resistant to further chemical alteration at higher intensities [25].

The observed increase in carbon content and corresponding decrease in oxygen content with rising laser intensity align with thermal degradation mechanisms widely reported for polymer textiles. Weclawski et al. (2021) [26] discovered similar elemental composition shifts when exposing Nylon 6.6 fabrics to combined atmospheric plasma/UV laser treatments, reporting oxygen depletion and carbon enrichment via XPS analysis. Their work indicates that high-energy exposure creates reactive surface species and promotes dehydroxylation, leading to a higher C/O ratio. In the present study, this trend plateaus at 225.4 × 10^6^ W/m^2^, suggesting that beyond this threshold, a thermally stable carbonaceous layer forms on the fabric surface, resisting further compositional change. This saturation behavior has been documented in laser carbonization studies of polyacrylonitrile nanofibers, where Go et al. (2016) [27] found that excessive laser fluence produces amorphous carbon layers with limited chemical transformation at higher intensities.

The mechanism underlying carbon enrichment likely involves preferential volatilization of oxygen-containing functional groups during localized heating. Horrocks et al. (2022) [28] investigated atmospheric plasma/UV laser treatment on cotton and polyester fabrics and observed via XPS that oxidizing plasma atmospheres initially increase the surface oxygen content by forming carboxyl and hydroxyl groups, but subsequent high-energy UV exposure led to decarboxylation and oxygen loss, consistent with our XRF findings. The cotton component in our blended fabric contains abundant hydroxyl groups that undergo thermally induced dehydration and decarboxylation, releasing H_2_O and CO_2_, while the polyester component undergoes chain scission and ester bond cleavage and the liberation of oxygen-rich volatiles [29]. In this experimental work, carbonization clearly began at a laser treatment intensity of 146.4 × 10^6^ W/m^2^ (sample 5, Figure 3d), where polyester fibers underwent adhesion and began melting (the start of an amorphous carbon-rich surface layer was formed).

The net result of the carbonization process is surface carbonization—a photothermal process where the lower melting point of polyester is reached, which melts and re-solidifies with reduced oxygen content [30]. By contrast, cotton fibers partially char, as shown in Figure 1o, sample 14.

**Figure 5:**
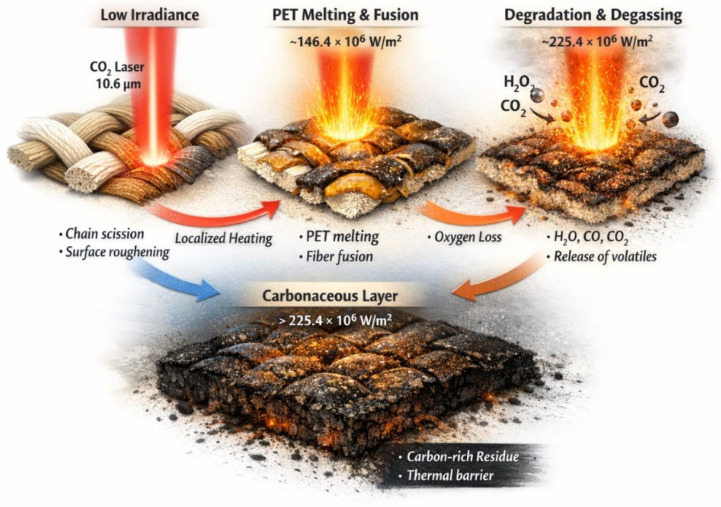
Schematic Representation of the Carbonization Process the surface of the fabric in continue of the laser treatment.

We clarified the mechanism of the carbonization process and identified the specific functional groups sensitive to CO_2_ laser treatment (Figure 5).

The carbonization (charring) induced by the CO_2_ laser can be described as a progressive thermal degradation of the fabric components (PET and Cotton):

– Initial Phase (Induction): At lower intensities, the laser energy causes the rupture of polymer chains and the initiation of surface roughening.
– Melting and Fusion (Intensity ~146.4 × 10^6^ W/m^2^): The polyester (PET) component, having a lower melting point, begins to melt and coat the cotton fibers, creating a carbon-rich amorphous layer.
– Degradation and Degassing: High thermal energy triggers the decomposition of oxygen-containing functional groups, releasing volatile species such as H_2_O, CO, and CO_2_.
– Formation of Carbonaceous Layer (Intensity up to 225.4 × 10^6^ W/m^2^): A stable carbon-rich residue (char) forms on the surface. This layer eventually acts as a thermal insulator, slowing down further deep chemical degradation as laser intensity increases.

Based on the FT-IR and WD-XRF analysis, the following functional groups are most sensitive to the laser treatment:

– Hydroxyl Groups (-OH): Abundant in the cellulose structure of cotton. These are highly sensitive to thermal energy and undergo dehydration and decarboxylation.
– Ester and Carbonyl Groups (C=O, C-O-C): Present in the polyester (PET) chains. The laser causes “chain scission” and the cleavage of ester bonds, leading to a significant loss of oxygen atoms.
– Methylene Groups (CH_2_): While less volatile, their relative exposure and orientation change as the polymer matrix rearranges during melting and carbonization.

To sum up the result, the laser treatment primarily affects the hydroxyl (–OH) groups in cotton and the ester/carbonyl groups (C=O, C-O-C) linkages in PET. The thermal energy causes the release of volatile components, leaving behind a carbon-rich surface layer, which is consistent with our WD-XRF data showing an increase in carbon content.

This interpretation contrasts somewhat with the findings of Ye et al. (2024) [31], who studied nanosecond laser thermal decomposition of carbon-fiber-reinforced plastics (CFRP) and reported that laser-induced graphitization could occur at sufficiently high fluences, leading to sp^2^ carbon formation. In our system, however, the absence of new chemical bonds in FT-IR spectra (discussed in Section 3.4) and the relatively moderate laser intensities employed suggest that full graphitization does not occur. Instead, the process depends on incomplete carbonization and the formation of amorphous carbon-rich surface layers. The plateau observed beyond 225.4 × 10^6^ W/m^2^ may reflect a balance between continued thermal input and the limited thermal conductivity of the partially carbonized surface, which acts as an insulating barrier preventing deeper material transformation. These findings underscore the importance of laser intensity selection for controlling surface chemistry in textile modification applications.

### 3.4. Laser Treatment Effect on Chemical Composition

**Figure 6:**
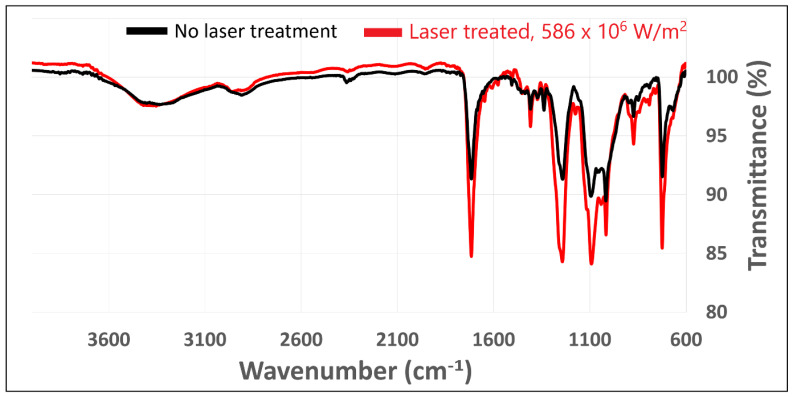
FT-IR spectra of untreated (black) and laser-treated (red) cotton/polyester/metal fabrics. The laser-treated sample 14 (Figure 1o) was processed at a power density of 586 × 10^6^ W/m^2^. Key spectral changes indicate modifications in chemical bonding and infrared absorption following CO_2_ laser exposure.

The FT-IR spectra of the untreated fabric and laser-treated sample 14 (Figure 1o, 586 × 10^6^ W/m^2^) for higher laser intensities are shown in Figure 6. Notable changes in absorption intensity are observed at wavenumbers 1715, 1241, 1094, 873, and 723 cm^−1^. These peaks correspond to specific vibrational modes associated with functional groups: C=O stretching (1715 cm^−1^), C–O–C stretching (1241 cm^−1^), C–O stretching (1094 cm^−1^), C=C bending (873 cm^−1^), and CH_2_ rocking (723 cm^−1^). The increase in IR absorption at these wavenumbers indicates that laser treatment enhances the presence or exposure of these chemical bonds [32].

These spectral changes suggest that CO_2_ laser irradiation leads to subtle chemical or structural rearrangements within the cotton/polyester/metal blend, resulting in reduced transparency in the infrared region. However, the absence of new peaks in the treated sample’s spectrum indicates that no major decomposition or formation of new functional groups occurred under the applied laser intensity.

The observed increase in peak intensities may also be attributed to changes in the physical structure of the fabric. Laser processing can soften or partially melt the fibers, improving the contact between the sample surface and the ATR crystal during measurement. This improved contact can lead to enhanced coupling of infrared radiation in the sample and, consequently, stronger absorption signals.

The absence of new chemical bonds in the FT-IR spectra, despite observable changes in peak intensities, indicates that CO_2_ laser treatment induces primarily physical rather than chemical modifications in the cotton/polyester/metal fabric. This finding is consistent with recent studies on laser-treated textiles. Horrocks et al. (2022) [28] reported that atmospheric plasma/UV laser treatment of cotton and polyester fabrics produced chemical changes that were not easily detected by FTIR-ATR, requiring more sensitive techniques such as XPS and electron paramagnetic resonance to identify new functional groups. Similarly, Weclawski et al. (2021) [26] found that while surface modifications occurred in Nylon 6.6 fabrics after combined plasma and UV laser exposure, FTIR-ATR proved insufficient for detecting subtle chemical alterations, suggesting that conventional infrared spectroscopy has inherent limitations in capturing surface-level chemical transformations. In the present study, the increased absorption at characteristic wavenumbers (1715, 1241, 1094, 873, and 723 cm^−1^) without the emergence of new peaks supports the interpretation that existing functional groups remained intact. As a result, intensity changes reflect physical reorganization rather than chemical decomposition.

The observed increase in FT-IR peak intensities can be attributed to improved contact between the sample and ATR, resulting from laser-induced melting and surface smoothing. During CO_2_ laser processing, the polyester component undergoes thermal softening and partial melting at temperatures well below its decomposition threshold [22]. This creates a more planar, consolidated surface that achieves better optical coupling with the diamond ATR crystal during measurement, thereby enhancing infrared radiation penetration and increasing the detected signal intensity [33]. Siwińska-Ciesielczyk et al. (2021) [34] conducted micro-FTIR analysis of heat-treated cotton/polyester blends and demonstrated that thermal exposure causes morphological changes, including polyester melting and fiber deformation, before significant chemical degradation occurs in either component. Their findings support the notion that physical restructuring precedes chemical alteration in thermally processed blended fabrics. The maintenance of characteristic polyester ester bonds (C=O at 1715 cm^−1^) and cotton cellulose bonds (C-O at 1094 cm^−1^) in our spectra confirms that the molecular backbone of fabrics is largely preserved despite surface morphological transformation.

Furthermore, the predominance of physical over chemical changes aligns with the moderate laser intensities employed in this study. Nandiyanto et al. (2023) [32] found that thermal degradation of polyesters typically begins with physical processes such as melting and viscosity reduction before progressing to chain scission and chemical decomposition at higher temperatures. The laser intensity range used here (19.1 × 10^6^ to 615.0 × 10^6^ W/m^2^) appears sufficient to induce surface melting and fiber fusion but insufficient to trigger extensive polymer degradation reactions that would generate new functional groups detectable by FTIR. This is further corroborated by the XRF results (Section 3.3), showing that elemental composition changes plateaued at 225.4 × 10^6^ W/m^2^. This suggests that there is a threshold beyond which the thermally modified surface layer acts as an insulating barrier. Recent work by Costa et al. (2024) [35] on cotton/polyester textile characterization via FTIR spectroscopy emphasized that reliable detection of chemical changes requires complementary techniques such as XPS or thermal analysis; FTIR alone may overlook surface-specific transformations. Collectively, these findings underscore that the observed spectral changes in our laser-treated fabrics reflect predominantly physical modifications, namely, improved ATR contact and surface consolidation, rather than fundamental chemical restructuring of the polymer matrix.

### 3.5. Laser Treatment Effect on Mechanical Properties

**Figure 7:**
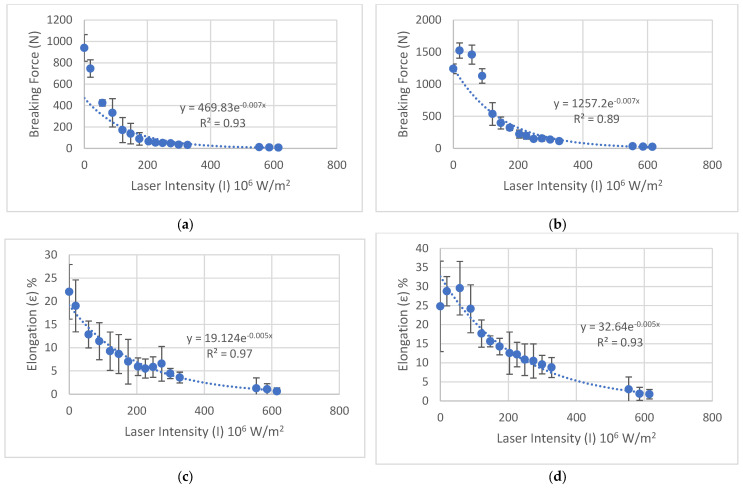
Mechanical properties of cotton/polyester/metal textile specimens treated with varying laser intensities. (**a**) Peak load for H series specimens, (**b**) Peak load for V series specimens, (**c**) elongation at peak load as a function of laser intensity for H series and (**d**) elongation at peak load as a function of laser intensity for V series, respectively. Each data point represents the mean of five measurements; error bars denote standard deviation.

The influence of laser treatment on the mechanical behavior of cotton/polyester fabrics is illustrated in Figure 7. The figure shows that increasing laser intensity results in a pronounced degradation of mechanical strength. Specifically, the peak load exhibits a substantial decrease as the applied laser intensity increases. This trend suggests that higher thermal input from the laser compromises the integrity of the fibrous network, likely due to thermal degradation, melting, or partial loss of structural cohesion in the polymer matrix.

A similar trend is observed for the elongation at peak load, as shown in Figure 7c,d, where ductility decreases progressively with laser intensity. The untreated reference specimen displayed the highest elongation, while specimens treated with intensities above 121 × 10^6^ W/m^2^ exhibited a marked reduction. At this threshold and beyond, peak load values decreased by approximately an order of magnitude, and elongation at peak load was reduced by nearly 50% compared to the untreated fabric.

These results indicate that CO_2_ laser processing significantly alters the mechanical performance of the textile, with intensity-dependent embrittlement occurring due to microstructural changes, such as fiber fusion, matrix stiffening, and localized material loss.

The results (Figure 7a,b) demonstrate a clear degradation in mechanical performance with increasing laser fluence. Data are derived from tensile testing in the warp direction only.

As illustrated in Figure 7a,b, increasing laser intensity results is a pronounced degradation of mechanical strength. The peak load exhibits a substantial decrease from approximately 800 N in the untreated specimen to below 100 N at intensities above 225 × 10^6^ W/m^2^. A similar trend is observed for the elongation at peak load (Figure 7c,d), where ductility decreases progressively with laser intensity, dropping from approximately 25% to below 5% at the highest treatment levels.

Dependence of mechanical properties of cotton/polyester/metal textile specimens treated with varying laser intensities can be approximated using power function. The solid lines in this graph correspond to the regression models with parameters obtained by the minimizing sum of squared errors. Corresponding coefficient of determination R2 = 0.95 indicates the good quality of fit.

Statistical analysis revealed significant inherent heterogeneity in the raw (untreated) material. The random error for the tensile strength measurements of the control samples along the weft (H0) reached 124 N (coefficient of variation is ≈13.2%), which is considerably higher than the error values observed for laser-treated materials. In contrast, for the samples tested along the warp (V0), the random error was 75 N (CV ≈ 6%), which is consistent with the average values for samples V1–V4 treated with laser intensities ranging from 19.1 to 121.0 × 106 W/m^2^. This high dispersion in the H0 group provides indirect evidence of the structural non-uniformity of the base material, which appears to be partially stabilized or homogenized by the laser treatment.

**Figure 8:**
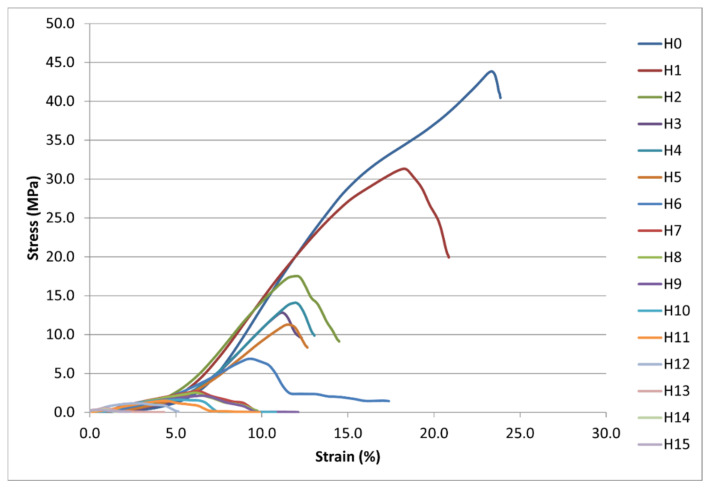
Stress–strain curves of hybrid fabric specimens tested in the weft direction (H-series, specimens 0–15).

**Figure 9:**
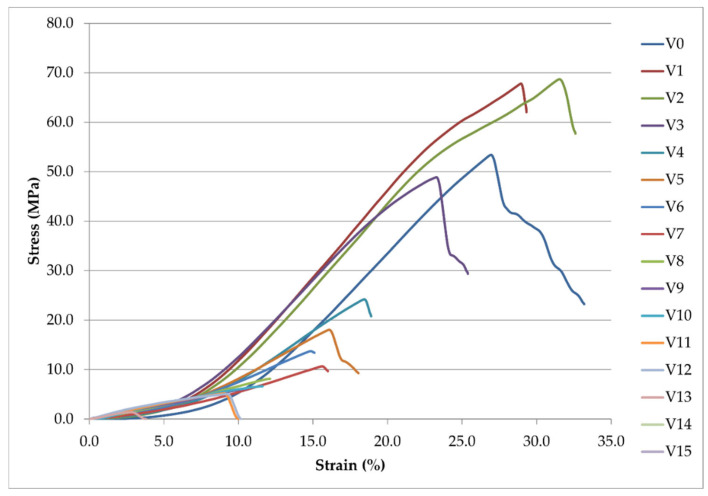
Stress–strain curves of hybrid fabric specimens tested in the warp direction (V-series, specimens 0–15).

To gain a deeper insight into the failure mechanisms and directional anisotropy of fabrics, complete stress–strain curves were analyzed for both warp (marked V-series) and weft (marked H-series) directions, as shown in Figure 8 and Figure 9, respectively.

The progressive reduction in ultimate tensile strength (UTS) and strain at break with increasing laser intensity demonstrates the severe mechanical degradation induced by CO_2_ laser treatment. The transition from ductile to brittle failure behavior is evident in the changing curve shapes.

Similarly to the weft direction, laser treatment causes a systematic decline in mechanical performance; however, the warp direction shows moderately higher absolute strength values due to the fabric weave structure.

To fully describe the mechanical properties, the average values of 10 measurements for each specimen are summarized in Table 2.

The stress–strain curves (Figure 8 and Figure 9) reveal distinct mechanical behavior across the laser intensity range. The untreated reference specimen exhibited typical ductile behavior with a well-defined yield point and substantial elongation before failure. In the weft direction (H-series), specimen 0 achieved an ultimate tensile strength (UTS) of 43.85 MPa with an elongation at break of 23.86%; in the warp direction (V-series), the UTS reached 53.38 MPa with 33.18% elongation (Table 2). This directional difference reflects the higher warp density (39 ends/cm) compared to the weft density (22 picks/cm) in the 2/1 twill weave structure, resulting in greater load-bearing capacity in the warp direction.

During laser treatment, the mechanical performance deteriorated progressively in both directions. Even at the lowest tested intensity (specimen 1, 19.1 × 10^6^ W/m^2^), significant changes were observed. By specimen 6 (146.0 × 10^6^ W/m^2^), the weft direction UTS had fallen to 6.86 MPa, which is an 84% reduction compared to the reference specimen. By contrast, the warp direction retained a relatively higher value of 13.73 MPa. At the highest intensities (specimens 13–15), both directions converged to similarly low UTS values below 2 MPa, indicating near-complete loss of mechanical integrity.

Young’s modulus (E) also decreased systematically with laser intensity in both directions (Table 2). In the weft direction, E declined from 347.76 MPa (specimen 0) to values below 70 MPa for moderately treated specimens (10–12). The warp direction displayed a similar pattern, with E decreasing from 317.81 MPa to approximately 56–115 MPa for heavily treated specimens.

Notably, elongation at break exhibited a non-monotonic response, which was particularly evident in the weft direction. After an initial decrease from 23.86% (specimen 0) to 12.32% (specimen 3), elongation paradoxically increased to 28.71% for specimen 7, before declining again to values below 5% for specimens 13–15. This temporary increase in elongation for mid-range intensities coincides with the morphological transition observed in SEM (Figure 3), where extensive polyester melting created a more homogeneous matrix capable of undergoing larger deformations before catastrophic failure, albeit at much lower stress levels.

The stress–strain curves (Figure 8 and Figure 9) also reveal a clear transition in failure modes across the laser intensity range. Low-intensity specimens (0–5) exhibited gradual necking and ductile tearing, characterized by a smooth stress plateau after the peak. Moderate-intensity specimens (6–10) had mixed brittle–ductile characteristics with irregular stress drops indicative of localized rupture of the fiber bundle. High-intensity specimens (11–15) failed due to their purely brittle characteristics. Results produced sharp stress peaks followed by immediate fracture and minimal post-yield deformation. This progression from ductile to brittle failure is consistent with the thermal degradation of the cotton fibers and the formation of a rigid, carbonized polyester-rich surface layer, as confirmed by XRF analysis (Figure 4) and FT-IR spectroscopy (Figure 6).

In this study, we investigated the effect of CO_2_ laser treatment on a 31 wt% cotton/59 wt% polyester blended fabric integrated with stainless-steel metal staple fibers (10 wt%). We focused on determining its chemical, elemental, morphological, optical, and mechanical properties.

A visual inspection showed that laser treatment induced progressive darkening of the fabric surface, transitioning from white to yellowish-brown. The optimal laser intensity range for controlled color modification was identified between 121 × 10^6^ W/m^2^ and 554.0 × 10^6^ W/m^2^.

High-resolution scanning electron microscopy (HR-SEM) revealed that increasing laser intensity led to surface melting and fiber fusion, particularly beyond 146 × 10^6^ W/m^2^. These changes resulted in the loss of the original fibrous structure and the formation of smoother, more consolidated surfaces. Similar surface transformations were reported by On-na Hung et al. [10].

Elemental analysis via wavelength-dispersive X-ray fluorescence (WD-XRF) showed a consistent increase in surface carbon content and a corresponding decrease in oxygen content with increasing laser intensity; this trend plateaued at 225.4 × 10^6^ W/m^2^. Complementary FT-IR spectroscopy confirmed that the chemical structure of the fabric was not significantly altered by the treatment, consistent with prior findings [12]. Notably, increased laser intensity was associated with more pronounced surface carbonization. Surface carbon contributes to enhanced absorption of visible light, reducing diffuse reflectance—particularly in the blue region—due to the optical behavior of the broadband of amorphous carbon. This mechanism explains the yellow-brown tint observed for the partially carbonized fabric [36].

These findings suggest that the laser predominantly melts the polyester component, forming a thermally stable composite with the cotton yarn.

Tensile testing showed substantial degradation in mechanical performance with increasing laser fluence. Even the lowest tested intensity (19.1 × 10^6^ W/m^2^) reduced the peak load by approximately 20%, while intensities above 121 × 10^6^ W/m^2^ led to a nearly tenfold reduction. The increase in stiffness and reduction in elongation are consistent with thermally induced embrittlement, as reported by On-na Hung et al. [22].

Overall, this study provides a detailed understanding of the structural and functional modifications induced by CO_2_ laser treatment in cotton/polyester/metal fabrics. The results establish key intensity thresholds beyond which material performance is significantly compromised. These insights are valuable for optimizing laser-based surface modification strategies in textile finishing, functionalization, and recycling.

Future work can build on the finding that the polyester remains chemically stable and forms a well-adhered molten layer during laser irradiation. This behavior may be exploited to embed nanoparticles or nanofibers into the fabric surface during laser processing (e.g., specimen 5 in Figure 3d, which had a relatively smooth fabric surface compared to other laser-treated specimens studied), enabling the fabrication of nanocomposite layers with tailored mechanical, electrical, or optical properties. Engineered surfaces could be developed for applications such as camouflage, providing enhanced stealth functionality in military textiles in the infrared radiation range.

### 3.6. Surface Wettability and Contact Angle Analysis

The wettability of the fabric surface was evaluated to understand the impact of laser treatment on the surface energy and fluid interaction of the PET/CO/SS blend. The results of the static water contact angle (WCA) measurements are summarized in Table 3.

For the untreated (control) fabric, the contact angle was found to be 105° ± 3°, which is typical for polyester-rich blends [37]. As the laser intensity increased from 19.1 × 106 to 615.0 × 106 W/m^2^, a progressive increase in the contact angle was observed, reaching a maximum of 135° ± 6° at the highest intensity.

This transition towards a more hydrophobic state can be attributed to two synergistic factors.

Chemical modification: As indicated by the WD-XRF analysis, laser treatment leads to a reduction in oxygen-containing functional groups (deoxidation) on the surface. The decrease in polar groups, such as the hydroxyl groups (–OH) associated with the cotton fibers, reduces the surface’s ability to form hydrogen bonds with water droplets.

Morphological changes: High-intensity laser radiation induces the thermal fusion of PET fibers and the removal of protruding cotton micro-fibrils. This process creates a redistributed surface roughness and a ‘melted’ polymer film that traps air pockets beneath the water droplet (the Cassie–Baxter model), significantly increasing the apparent contact angle.

To sum up the result, the measurements revealed that laser treatment increases the water contact angle from 105° to 135°. This trend correlates with the WD-XRF data, suggesting that the reduction in surface oxygen and the thermal reorganization of the polymer fibers lead to a more hydrophobic surface characteristic.

## 4. Conclusions

This study systematically investigated the effects of CO_2_ laser treatment on cotton/polyester/metal blended fabric across laser intensities ranging from 19.1 × 10^6^ to 615.0 × 10^6^ W/m^2^. The results demonstrate that laser treatment induces progressive surface darkening through thermal carbonization, with visible color changes occurring above 121.0 × 10^6^ W/m^2^ and plateauing beyond 554.0 × 10^6^ W/m^2^. Elemental analysis revealed carbon enrichment and oxygen depletion up to 225.4 × 10^6^ W/m^2^, which is consistent with the thermally driven dehydroxylation and volatilization of oxygen-containing groups. FT-IR spectroscopy confirmed that these changes are predominantly physical rather than chemical, with no new functional groups formed, while SEM imaging showed progressive fiber fusion and surface smoothing due to polyester melting.

Mechanical testing revealed substantial degradation in fabric performance, with the peak load decreasing by approximately 90% and elongation reducing by 50% at laser intensities above 121 × 10^6^ W/m^2^. The stress–strain analysis demonstrated a transition from ductile to brittle failure behavior, indicating thermal embrittlement of the fibrous structure. The warp direction consistently exhibited higher mechanical resilience than the weft direction due to the fabric’s 2/1 twill weave structure, though both directions converged with similarly degraded properties at the highest intensities.

These findings establish critical laser intensity thresholds for cotton/polyester/metal fabric modification: an intensity below 121 × 10^6^ W/m^2^ should be used for minimal mechanical degradation; an intensity between 121 and 225 × 10^6^ W/m^2^ should be used for controlled surface modification with acceptable property retention; and intensities above 225 × 10^6^ W/m^2^ should be used when severe embrittlement limits practical applications. Future work should explore embedding functional nanoparticles during laser processing to create nanocomposite surfaces with enhanced optical, electrical, and protective properties for military textile applications such as infrared camouflage.

## Figures and Tables

**Table 1:** Laser intensities used to treat the 15 fabric samples.

Sample Number	Laser Intensity (I) 10^6^ W/m^2^	Sample Number	Laser Intensity (I) 10^6^ W/m^2^	Sample Number	Laser Intensity (I) 10^6^ W/m^2^
1	19.1	6	174.4	11	299.2
2	57.3	7	203.7	12	327.2
3	89.1	8	225.4	13	554.0
4	121.0	9	248.3	14	586.0
5	146.4	10	273.7	15	615.0

**Table 2:** Mechanical properties of hybrid fabric specimens in the warp (V-series) and weft (H-series) directions as a function of laser treatment intensity (all measurements are within a ±95%confidence interval).

Name	Breaking Load (F) N ISO 13934-1:2013	Elongation at Break (%) ISO 13934-1:2013	Thickness (mm)	E (MPa)	UTS (MPa)
H0	939 ± 124	22.04 ± 6	0.46 ± 0.01	347.76	43.85
H1	746 ± 81	19 ± 6	0.46 ± 0.01	302.44	31.33
H2	425 ± 29	12.84 ± 3	0.46 ± 0.01	266.17	17.53
H3	332.8 ± 132	11.4 ± 4	0.46 ± 0.01	225.24	12.81
H4	171.58 ± 117	9.24 ± 4	0.46 ± 0.01	221.80	14.10
H5	137.74 ± 96	8.64 ± 4	0.47 ± 0.01	178.23	17.65
H6	88.98 ± 58	7 ± 5	0.48 ± 0.01	121.37	6.86
H7	65.24 ± 15	5.92 ± 2	0.48 ± 0.01	43.42	2.71
H8	54.2 ± 5	5.52 ± 2	0.48 ± 0.01	41.66	2.51
H9	51.32 ± 4	5.84 ± 2	0.49 ± 0.01	25.51	2.13
H10	47.96 ± 8	6.56 ± 4	0.49 ± 0.01	61.56	1.6
H11	35.33 ± 2	4.44 ± 1	0.50 ± 0.01	39.41	1.46
H12	32.46 ± 3	3.6 ± 1	0.50 ± 0.01	66.92	1.14
H13	11.904 ± 2	1.28 ± 2	0.50 ± 0.01	-	0.49
H14	9.074 ± 3	1.08 ± 1	0.49 ± 0.01	-	0.59
H15	7.872 ± 2	0.64 ± 1	0.49 ± 0.01	-	0.47
V0	1240 ± 75	24.8 ± 12	0.46 ± 0.01	317.81	53.38
V1	1524 ± 118	28.76 ± 4	0.46 ± 0.01	352.17	67.80
V2	1460 ± 149	29.56 ± 7	0.46 ± 0.01	343.02	68.66
V3	1128.6 ± 114	24.15 ± 6	0.46 ± 0.01	317.75	48.87
V4	536.6 ± 176	17.64 ± 4	0.46 ± 0.01	210.34	24.2
V5	396 ± 93	15.6 ± 1	0.47 ± 0.01	164.21	28.25
V6	322.4 ± 42	14.24 ± 2	0.48 ± 0.01	115.63	13.73
V7	224.2 ± 62	12.52 ± 6	0.48 ± 0.01	97.09	10.65
V8	195 ± 49	12.16 ± 3	0.48 ± 0.01	62.90	8.12
V9	147.6 ± 31	10.8 ± 4	0.49 ± 0.01	58.49	6.10
V10	155.6 ± 41	10.48 ± 4	0.49 ± 0.01	60.57	6.66
V11	137.2 ± 15	9.52 ± 2	0.50 ± 0.01	61.39	5.23
V12	113.34 ± 19	8.76 ± 3	0.50 ± 0.01	56.47	5.06
V13	34.32 ± 12	3.04 ± 3	0.50 ± 0.01	67.64	1.40
V14	27.66 ± 14	1.88 ± 2	0.49 ± 0.01	115.41	1.78
V15	25.42 ± 9	1.76 ± 1	0.49 ± 0.01	58.26	1.34

**Table 3:** Static water contact angle of the fabric at different laser intensities.

Sample Number	Laser Intensity (10^6^ W/m^2^)	Contact Angle, θ (Degrees)	Surface State
Control	0	105° ± 3°	Hydrophobic
1	19.1	108° ± 4°	Hydrophobic
4	121.0	115° ± 3°	Hydrophobic
8	225.4	128° ± 5°	Highly Hydrophobic
15	615.0	135° ± 6°	Highly Hydrophobic

## Data Availability

Data is contained within the article.

## References

[1] Wang X., Shen X., Xu W. (2012). Effect of hydrogen peroxide treatment on the properties of wool fabric. Appl. Surf. Sci..

[2] Strobel M., Walzak M.J., Hill J.M., Lin A., Karbashewski E., Lyons C.S. (1995). A comparison of gas-phase methods of modifying polymer surfaces. J. Adhes. Sci. Technol..

[3] Sanchaniya J.V., Lasenko I., Kanukuntla S.P., Mannodi A., Viluma-gudmona A., Gobins V. (2023). Preparation and Characterization of Non-Crimping Laminated Textile Composites Reinforced with Electrospun Nanofibers. Nanomaterials.

[4] Sanchaniya J.V., Lasenko I., Vijayan V., Smogor H., Gobins V., Kobeissi A., Goljandin D. (2024). A Novel Method to Enhance the Mechanical Properties of Polyacrylonitrile Nanofiber Mats: An Experimental and Numerical Investigation. Polymers.

[5] Sanchaniya J.V., Lasenko I., Kanukuntala S.P., Smogor H., Viluma-Gudmona A., Krasnikovs A., Tipans I., Gobins V. (2023). Mechanical and Thermal Characterization of Annealed Oriented PAN Nanofibers. Polymers.

[6] Kan C.-W., Lo C.K.Y., Man W.S. (2016). Environmentally friendly aspects in coloration. Color. Technol..

[7] Sanchaniya J.V., Lasenko I., Gobins V., Kobeissi A. (2024). A Finite Element Method for Determining the Mechanical Properties of Electrospun Nanofibrous Mats. Polymers.

[8] Lasenko I., Sanchaniya J.V., Kanukuntla S.P., Viluma-Gudmona A., Vasilevska S., Vejanand S.R. (2024). Assessment of Physical and Mechanical Parameters of Spun-Bond Nonwoven Fabric. Polymers.

[9] Gulbinienė A., Fataraitė-Urbonienė E., Jucienė M., Dobilaitė V., Valeika V. (2021). Effect of CO2 laser treatment on the leather surface morphology and wettability. J. Ind. Text..

[10] Gaffar M.A., Ahammed B., Hoque T., Maksura S., Islam M.R., Shahid M., Maiti S., Khan S.A., Adivarekar R.V. (2025). Advanced Surface Modification Techniques for Sustainable Coloration of Textile Materials. Advancements in Textile Coloration: Techniques, Technologies, and Trends.

[11] Gupta P., Shukla A., Shahid M., Biranje S., Yusuf M., Adivarekar R. (2025). Advancements in Plasma Technology for Textile Surface Modification. Advancements in Textile Finishing: Techniques, Technologies, and Trends.

[12] Ekunde R.A., Sutar R.S., Ingole S.S., Jundle A.R., Gaikwad P.P., Saji V.S., Liu S., Bhosale A.K., Latthe S.S. (2025). Fluorine-free and breathable self-cleaning superhydrophobic CS/PDMS/PLA coatings on wearable cotton fabric with light-induced self-sterilization. Prog. Org. Coat..

[13] Kan C.W. (2008). Impact on textile properties of polyester with laser. Opt. Laser Technol..

[14] Chow Y.L., Chan C.K., Kan C.W. (2011). Effect of CO2 laser treatment on cotton surface. Cellulose.

[15] Chow Y.L.F., Chan A., Kan C.-W. (2011). Effect of CO2 laser irradiation on the properties of cotton fabric. Text. Res. J..

[16] Hung O.N., Chan C.K., Kan C.W., Yuen C.W.M., Song L.J. (2014). Artificial neural network approach for predicting colour properties of laser-treated denim fabrics. Fibers Polym..

[17] Kan C. (2014). CO2 laser treatment as a clean process for treating denim fabric. J. Clean. Prod..

[18] Kan C.W. (2014). Colour fading effect of indigo-dyed cotton denim fabric by CO2 laser. Fibers Polym..

[19] Kukle S., Lazov L., Lohmus R., Briedis U., Adijans I., Bake I., Dunchev V., Teirumnieka E. (2025). The Impact of CO2 Laser Treatment on Kevlar® KM2+ Fibres Fabric Surface Morphology and Yarn Pull-Out Resistance. Polymers.

[20] Bormashenko E., Pogreb R., Sheshnev A., Shulzinger E., Bormashenko Y., Katzir A. (2001). IR laser radiation induced changes in the IR absorption spectra of thermoplastic and thermosetting polymers. J. Opt. A Pure Appl. Opt..

[21] Hung O.-N., Chan C.-K., Kan C.-W., Yuen C.-W. (2016). Marcus Microscopic study of the surface morphology of CO2 laser-treated cotton and cotton/polyester blended fabric. Text. Res. J..

[22] Hung O., Kan C. (2017). Effect of CO2 Laser Treatment on the Fabric Hand of Cotton and Cotton/Polyester Blended Fabric. Polymers.

[23] Hung O.N., Chan C.K., Kan C.W., Yuen C.W.M. (2017). An analysis of some physical and chemical properties of CO2 laser-treated cotton-based fabrics. Cellulose.

[24] Hung O., Kan C. (2017). A Study of CO2 Laser Treatment on Colour Properties of Cotton-Based Fabrics. Coatings.

[25] Jucienė M., Urbelis V., Juchnevičienė Ž., Čepukonė L. (2013). The effect of laser technological parameters on the color and structure of denim fabric. Text. Res. J..

[26] Weclawski B.T., Horrocks A.R., Ebdon J.R., Mosurkal R., Kandola B.K. (2021). Combined atmospheric pressure plasma and UV surface functionalisation and diagnostics of nylon 6.6 fabrics. Appl. Surf. Sci..

[27] Go D., Lott P., Stollenwerk J., Thomas H., Möller M., Kuehne A.J.C. (2016). Laser Carbonization of PAN-Nanofiber Mats with Enhanced Surface Area and Porosity. ACS Appl. Mater. Interfaces.

[28] Ayesh M., Horrocks A.R., Kandola B.K. (2022). The Impact of Atmospheric Plasma/UV Laser Treatment on the Chemical and Physical Properties of Cotton and Polyester Fabrics. Fibers.

[29] Chen H., Chen F., Chen H., Liu H., Chen L., Yu L. (2022). Thermal degradation and combustion properties of most popular synthetic biodegradable polymers. Waste Manag. Res..

[30] Ma T., Wang W., Wang R. (2023). Thermal Degradation and Carbonization Mechanism of Fe-Based Metal-Organic Frameworks onto Flame-Retardant Polyethylene Terephthalate. Polymers.

[31] Ye Y., Yuan Z., Zhang Z., Chen G., Pei G., Guo F., Ren X. (2024). Experimental study on nanosecond laser thermal decomposition of CFRP and recycling of carbon fibers. Opt. Laser Technol..

[32] Bayu A., Nandiyanto D., Ragadhita R., Fiandini M. (2023). Interpretation of Fourier Transform Infrared Spectra (FTIR): A Practical Approach in the Polymer/Plastic Thermal Decomposition. Indones. J. Sci. Technol..

[33] Irwan I., Mohamad J.N., Galih V., Putra V. (2023). FT-IR Spectral model of polyester-cotton fabrics with corona plasma treatment using artificial neural networks (ANNs). Indones. J. Appl. Phys..

[34] Machnowski W., Wąs-Gubała J. (2021). Evaluation of Selected Thermal Changes in Textile Materials Arising in the Wake of the Impact of Heat Radiation. Appl. Sci..

[35] Paz M.L., Sousa C. (2024). Discrimination and Quantification of Cotton and Polyester Textile Samples Using Near-Infrared and Mid-Infrared Spectroscopies. Molecules.

[36] Delacroix S., Wang H., Heil T., Strauss V. (2020). Laser-Induced Carbonization of Natural Organic Precursors for Flexible Electronics. Adv. Electron. Mater..

[37] Khalili Gashtroudkhani A., NazarpourFard H., Ghobadian A., Alihoseini M. (2024). Non-thermal, atmosphere pressure and roll-to-roll plasma treatment for improving the surface characteristics and antistatic solution absorption in polyester fabrics. J. Text. Inst..

